# Prognostic effect of isolated paraaortic nodal spread in endometrial cancer

**DOI:** 10.4274/jtgga.2017.0152

**Published:** 2018-11-15

**Authors:** Osman Türkmen, Derman Başaran, Alper Karalök, Günsu Cömert Kimyon, Tolga Taşçı, Işın Üreyen, Gökhan Tulunay, Taner Turan

**Affiliations:** 1 Clinic of Gynecologic Oncology, Gaziantep Cengiz Gökçek Obstetrics and Children’s Hospital, Gaziantep, Turkey; 2 Clinic of Gynecologic Oncology, University of Health Sciences, Etlik Zübeyde Hanım Women’s Health Training and Research Hospital, Ankara, Turkey; 3 Clinic of Gynecologic Oncology, İstanbul Okmeydanı Training and Research Hospital, İstanbul, Turkey; 4 Clinic of Gynecologic Oncology, University of Health Sciences, Antalya Training and Research Hospital, Antalya, Turkey; 5 Clinic of Gynecologic Oncology, TOBB University Hospital, Ankara, Turkey

**Keywords:** Endometrial cancer, paraaortic lymph node metastasis, serosal involvement

## Abstract

**Objective:**

To evaluate the prognostic effect of isolated paraaortic lymph node metastasis in endometrial cancer (EC).

**Material and Methods:**

This retrospective study included patients with FIGO 2009 stage IIIC2 disease due to isolated paraaortic lymph node metastasis (LNM). Patients with sarcomatous histology, synchronous gynecologic cancers and patients with concurrent pelvic lymph node metastases or patients that have intraabdominal tumor spread were excluded. Kaplan-Meier method was used for calculation of progression free survival (PFS) and overall survival.

**Results:**

One thousand six hundred and fourteen patients were operated for EC during study period. Nine hundred and sixty-one patients underwent lymph node dissection and 25 (2.6%) were found to have isolated LNM in paraaortic region and these constituted the study cohort. Twenty (80%) patients had endometrioid EC. Median number of retrieved lymph nodes from pelvic region and paraaortic region was 21.5 (range: 5-41) and 34.5 (range: 1-65), respectively. Median number of metastatic paraaortic nodes was 1 (range: 1-32). The median follow-up time was 15 months (range 5-94). Seven (28%) patients recurred after a median of 20 months (range, 3-99) from initial surgery. Three patients recurred only in pelvis, one patient had upper abdominal spread and 3 had isolated extraabdominal recurrence. Involvement of uterine serosa, positive peritoneal cytology and presence of adnexal metastasis were significantly associated with diminished PFS (p<0.05).

**Conclusion:**

The presence of serosal involvement or adnexal involvement is as important as gross peritoneal spread and is related with poor survival in patients with isolated paraaortic nodal spread in EC. Chemotherapy should be the mainstay of treatment in this patient cohort which may eradicate systemic tumor spread.

## Introduction

Endometrial cancer (EC) is the most common gynecologic malignancy and the 4^th^ most common cancer of women (1). Surgical staging of EC was recommended by the International Federation of Gynecology and Obstetrics (FIGO) in 1988 and has been re-validated in the most recent FIGO 2009 staging system (2,3). One of the most striking differences in the FIGO 2009 staging system was the division of the former stage IIIC of the FIGO 1988 system into two subgroups; stage IIIC1 and IIIC2. Patients with only positive pelvic lymphatic spread were defined as stage IIIC1 and those with paraaortic lymphatic spread irrespective of pelvic lymph nodes were named as stage IIIC2 (4). However, patients with stage IIIC2 disease constitute a very heterogeneous patient cohort in whom either pelvic extrauterine disease or positive pelvic lymph nodes may exist concurrently. Another important patient subgroup of FIGO stage IIIC2 disease, with an incidence varying between 1% and 6% among all patients with EC, comprises patients with isolated paraaortic lymph node metastases (5,6,7,8,9). Tumors of the uterine corpus may spread to the paraaortic region via lymphatic routes of obturator and external iliac chains or the lymphatic pathways through gonadal vessels (10). These findings indicate that even patients with stage IIIC2 disease due to isolated paraaortic lymph nodes may represent a heterogeneous patient group because of lymphatic spread patterns.

In this retrospective study we sought to define the clinical and surgical factors related to the prognosis of patients with isolated paraaortic lymph spread in EC.

## Material and Methods

This retrospective study included patients with epithelial EC who underwent comprehensive surgical staging at the Gynecologic Oncology Department of our hospital from January 1992 to September 2014. The inclusion criterion was having FIGO 2009 stage IIIC2 disease due to isolated paraaortic lymph node metastasis (LNM). Patients with sarcomatous histology, synchronous gynecologic cancers, and patients with concurrent pelvic LNMs or intraabdominal tumor spread were excluded. Demographics, intraoperative findings, and surgico-pathological results were accumulated from patient files, pathology reports, and electronic files.

All patients underwent hysterectomy and bilateral salpingo-oophorectomy and complete systematic lymph node dissection. Bilateral pelvic lymphadenectomy was performed to complete skeletonization, with all lymphatic tissue of the pelvic vascular structure and the obturator fossa. Between the aortic bifurcation and deep circumflex iliac vein, lymphatic tissues around the pelvic vascular structure and lymphatic tissue in obturator fossa were defined as pelvic lymph nodes. All lymphatic tissues around the vascular structure between the aortic bifurcation and renal vein were defined as para aortic lymph nodes. All surgical procedures were performed by gynecologic oncologists and pathologic evaluations were reported by a dedicated team of and gynecopathologists.

All patients received adjuvant treatment in forms of chemotherapy, radiotherapy or combined chemoradiation after initial surgery. The decision regarding the type of adjuvant radiotherapy was made by a gynecologic oncology council according to the patient’s risk factors. Chemotherapy regimens included platinum and taxane- based protocols. External beam radiotherapy (EBRT) was given to the patients. EBRT was directed to the pelvis in the para-aortic region. Pelvic radiotherapy targeted the lower common iliacs, external iliacs, internal iliacs, parametrium, upper vagina, and paravaginal tissue. Extended-field radiotherapy included pelvic tissues and the para-aortic lymph node region up to the renal vessels. The external beam doses were 45 to 50 Gy. Adnexal and uterine serosal involvement and positivity of peritoneal cytology were accepted as non-nodal extrauterine disease. Progression-free survival (PFS) was defined as the time between surgery and relapse or the last follow-up. The duration until exitus because of the disease or until the follow-up was defined as overall disease-specific survival (OS). We defined recurrence distal to the linea terminalis as pelvic recurrence, recurrence between the pelvic inlet and diaphragm peritoneum as upper abdominal recurrence, and other recurrences such as recurrence in the liver parenchyma, skin, and bone were accepted as extra-abdominal recurrence. Ascites and peritonitis carcinomatosa were accepted as upper abdominal recurrence.

After adjuvant treatment was completed, patients were followed up every three months for the first 2 years, then every 6 months for 3 years, and annually thereafter. The follow-up routine included pelvic inspection and full abdominal ultrason ography. Chest X-rays were performed yearly unless there was clinical suspicion. Thoracic and/or abdominal computerized tomography was performed when necessary. Ca-125 levels were used in the follow-up.

SPSS (SPSS Inc, Chicago IL, USA) version 15.0 was used for statistical analyses. Kaplan-Meier analysis was used for the calculation of PFS and OS. Survival curves were compared using the log-rank test. Prognostic factors were evaluated using the Cox regression model. The cut-off for statistical significance was set at p<0.05. A multivariate model was not created because of the intercorrelations between parameters that were significant in the univariate analysis. This study was approved by the local ethics committee. Signed informed consent was given by all patients, which allowed us to use their clinical data.

## Results

One thousand six hundred fourteen patients underwent surgery for EC during the study period. Of these, 42 patients were excluded because of having synchronous cancers. Nine hundred sixty-one out of 1572 patients underwent lymph node dissection and 25 (2.6%) were found to have isolated LNM in the paraaortic region, and these constituted the study cohort. The median age of the patients was 60 years (range, 44-77 years). The surgical and pathologic features of the patients are presented in Table 1. Twenty (80%) patients had endometrioid type of EC and 19 (76%) had lymphovascular space invasion in paraffin histologic sections. The median number of removed lymph nodes from the pelvic area and paraaortic area was 21.5 (range, 5-41) and 34.5 (range, 1-65), respectively. The median number of metastatic paraaortic nodes was 1 (range, 1-32).

All patients with isolated paraaortic LNM received adjuvant treatment; 14 had radiotherapy, 6 received chemotherapy, and 5 underwent combined chemoradiation (sandwich protocol). Complete response was documented in all patients after adjuvant treatment. The median follow-up time was 15 months (range, 5-94). Seven (28%) patients had recurrence after a median of 20 months (range, 3-99) from the initial surgery. Three patients had recurrence only in the pelvis, one patient had upper abdominal spread, and 3 had isolated extraabdominal recurrence. Two (8%) patients died of disease.

Univariate analysis showed that uterine serosal involvement, positivity of peritoneal cytology, presence of adnexal metastasis and presence of non-nodal extrauterine disease were significantly associated with diminished PFS (Table 2, 3). The median OS was 15 months. Only lymphovascular space invasion but not age, uterine serosal invasion, adnexal metastasis, non-nodal extrauterine disease, tumor histology, myometrial invasion, grade, type of adjuvant treatment, number of metastatic paraaortic lymph nodes, cervical stromal/glandular invasion and preoperative CA125 level were found to be associated with OS.

## Discussion

The incidence of paraaortic LNM in EC and the prognostic factors of patients with stage IIIC2 EC have been the subject of various studies in the literature; however, there are limited data on the prognosis of isolated paraaortic LNM in EC (5,6,7,8,9,11,12). Todo et al. (13) conducted a study to evaluate the prognosis of stage IIIC EC. A total of 93 patients with stage IIIC EC were classified into three groups: group 1 consisted of patients who underwent pelvic and paraaortic lymphadenectomy and were positive for pelvic LNM (stage IIIC1), group 2 underwent only pelvic lymphadenectomy and had positive pelvic spread (at least stage IIIC1), and patients in group 3 had positive paraaortic lymph nodes (stage IIIC2). The 5-year survival rates were 89.3% in group 1, 46.5% in group 2, and 59.9% in group 3. Group 2 (p=0.0001) and group 3 (p=0.0016) had significantly diminished overall survival rates compared with group 1. They showed that metastatic lymph node count, lymphadenectomy level, and type of adjuvant therapy were significantly and independently associated with overall survival. Marchetti et al. (14) showed that a combined approach with chemotherapy and radiotherapy might improve recurrence-free survival compared with radiation or chemotherapy alone for stage IIIC EC. In the present study, metastatic node count in the paraaortic region and adjuvant treatment modality options were not related with PFS in patients with isolated paraaortic LNM (p=0.056 and p=0.967, respectively).

The prognostic role of uterine factors in stage IIIC EC was evaluated by Hoekstra et al. (15). Their study included 54 patients with pelvic spread and 31 patients with paraaortic (±pelvic) spread. In multivariate analysis, age, clear cell and serous h istology, and invasion rate were significantly related with OS; age and non-endometrioid histology were related with PFS. In the present study, presence of deep myometrial invasion (except serosal invasion) and non-endometrioid histology was not related with diminished survival. This finding is probably the result of the homogeneity of patients with isolated paraaortic LNM in our study because depth of myometrial invasion and non-endometrial histology are proved factors for lymphatic spread (16,17).

The significance of extranodal disease in stage IIIC EC was evaluated in a Surveillance, Epidemiology, and End Results (SEER) database analysis by Garg et al. (18). In patients with IIIC2 disease, high-risk factors such as grade III disease (p<0.001), non-endometrioid histologic types (p=0.01), and extrauterine disease (p<0.001) were common when compared with patients with stage IIIC1 disease. However, further analysis showed that in patients with extranodal disease, area of nodal metastasis had no effect on survival (HR=0.92; 95% CI: 0.74-1.14) and patients with positivity of peritoneal cytology and adnexal/serosal metastasis. These results support our findings that patients with isolated paraaortic LNM had unfavorable survival in the positivity of extranodal disease. Serosal involvement (p=0.007), positivity of peritoneal cytology (p=0.002), and adnexal metastasis (p=0.0021) were related with worse survival in our study. When the patients were classified into two groups according to the presence of extranodal disease, patients with no extranodal disease had significantly better outcomes (p=0.002). Therefore, one can conclude that patients with isolated paraaortic nodes have poor survival when the disease spreads intraperitoneally. 

The major limitation of the current study is the inherent drawbacks from its retrospective design. However, histologic examination by experienced gynecologic pathologists, expert gynecologic oncologists, the high lymph node counts from all node bearing regions, and the relatively high number of cases with isolated paraaortic LNM are some strong sides of this study.

In our study, only lymphovascular space invasion was associated with OS. Lymphovascular space invasion (LVSI) has two components. In our cohort, all patients had lymphatic spread so a lymphatic component of LVSI was present in all patients. Four of the seven recurrences were out of the lymphatic region. The vascular component of LVSI is associated with extraabdominal recurrence and upper abdominal recurrence (19). The effect of LVSI on OS may occur because of the vascular component of LVSI.

In conclusion, our stu dy showed that peritoneal spread including adnexal metastasis, uterine serosal involvement, and positive peritoneal cytology rather than tumor histology or uterine factors determine the prognosis in patients with isolated positive paraaortic lymph nodes in EC.

## Figures and Tables

**Table 1:**
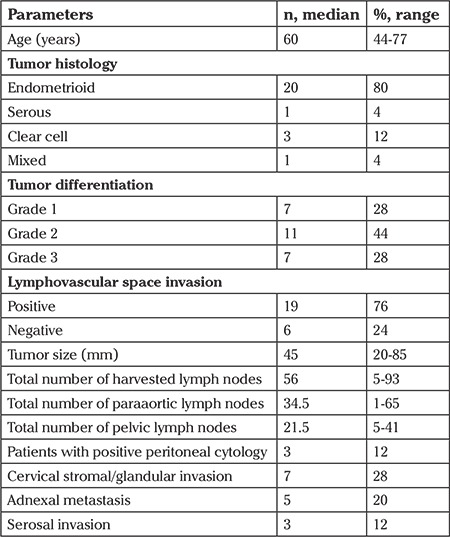
Characteristics of the patients with isolated paraaortic lymph node metastasis (n=25)

**Table 2:**
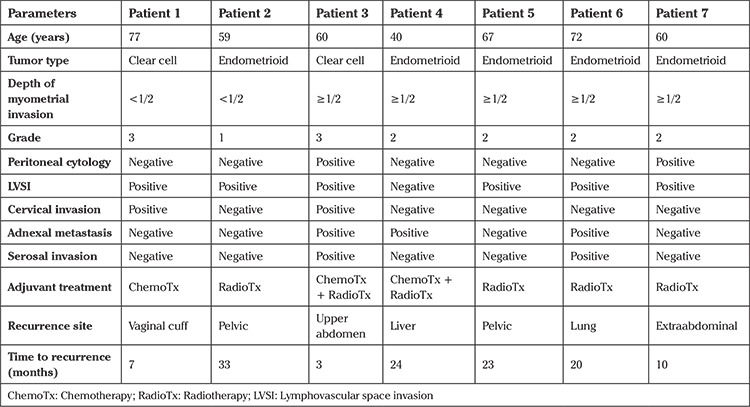
Characteristics of the patients with recurrent disease

**Table 3:**
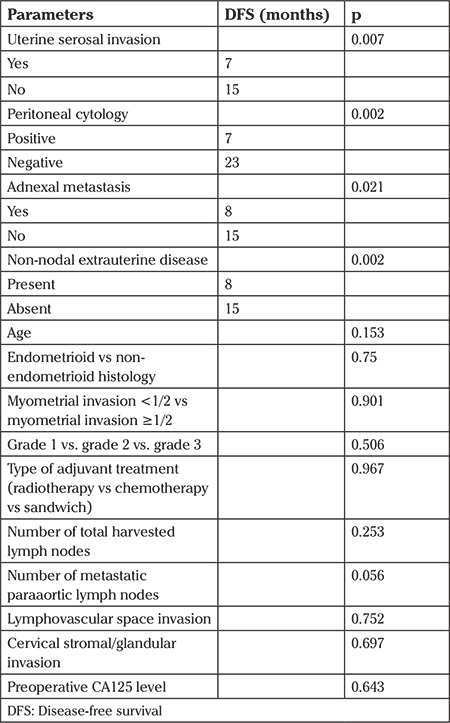
Univariate analysis of surgical factors associated with disease-free survival
